# Dose of sucrose affects the efficacy of Qiweibaizhu powder on antibiotic-associated diarrhea: Association with intestinal mucosal microbiota, short-chain fatty acids, IL-17, and MUC2

**DOI:** 10.3389/fmicb.2023.1108398

**Published:** 2023-01-19

**Authors:** Cuiru Li, Nenqun Xiao, Na Deng, Dandan Li, Zhoujin Tan, Maijiao Peng

**Affiliations:** ^1^College of Chinese Medicine, Hunan University of Chinese Medicine, Changsha, China; ^2^College of Pharmacy, Hunan University of Chinese Medicine, Changsha, China

**Keywords:** Qiweibaizhu powder, sucrose, antibiotic-associated diarrhea, intestinal mucosal microbiota, short-chain fatty acids

## Abstract

**Introduction:**

Due to the poor taste of Qiweibaizhu powder (QWBZP), patients have difficulty taking medicine, which leads to poor compliance and limits clinical use to a certain extent. In the trend of restricting sugar intake, sweeteners have gained massive popularity, among which sucrose is a commonly used sweetener in preparations. This study aimed to investigate the effect of different sucrose dose addition with antibiotic-associated diarrhea (AAD) by intervened QWBZP on intestinal mucosal microbiota.

**Methods:**

Thirty specific-pathogen-free (SPF) Kunming (KM) male mice were randomly divided into normal group (N), natural recovery group (M), QWBZP group (Q), low dose sucrose group (LQ), medium dose sucrose group (MQ), and high dose sucrose group (HQ). Subsequently, 16S rRNA amplicon sequencing and GC-MS techniques were used to analyze the intestinal mucosal microbiota and short-chain fatty acid (SCFAs) in intestinal contents, respectively, and enzyme-linked immunosorbent assay was used to determine mucin 2 (MUC2) and interleukin 17 (IL-17).

**Results:**

Compared with the Q group, the results showed that with the increase of sucrose dose, the intestinal microbial structure of mice was significantly altered, and the intestinal microbial diversity was elevated, with the poor restoration of the intestinal biological barrier, decreased content of SCFAs, high expression of inflammatory factor IL-17 and decreased content of mucosal protective factor MUC2. In conclusion, we found that the addition of sucrose had an effect on the efficacy of the AAD intervented by QWBZP, which was less effective than QWBZP, showing a certain dose-response relationship. In this experiment, it was concluded that the addition of sucrose might also further lead to intestinal inflammation and the disruption of the intestinal mucosal barrier, and the production of metabolites SCFAs.

**Discussion:**

The addition of sucrose might also further lead to intestinal inflammation and the disruption of the intestinal mucosal barrier, and the production of metabolites SCFAs. However, these findings still need to be verified in a more extensive study. The effect of adding the sweetener sucrose on the efficacy of Chinese herbal medicine in treating diseases also still needs more research.

## 1. Introduction

Antibiotic-associated diarrhea (AAD) is a medical diarrhea of varying severity caused by the application of antibiotics without a clear etiology and resulting in the suppression of sensitive intestinal bacteria and the proliferation of non-sensitive bacteria. At the same time, other etiologies (irritable bowel syndrome, inflammatory bowel disease, food allergy, etc.) need to be excluded (Yuan, 2021). According to the World Health Organization, billions of cases of diarrhea occur worldwide each year, with infants and young children being the main group affected. The treatment of pediatric diarrheal diseases in China suffers from the misuse of antimicrobials, and the increase in adverse reactions due to the irrational use of antibiotics is one of the prominent representatives of AAD. Nowadays, antibiotics are widely used, destroying pathogenic bacteria while killing many normal bacteria in the intestinal tract, thus causing dysbiosis of the intestinal microbiota. It further disrupts the metabolic capacity of the organism, thus slowing down the development of the microbial community, impairing the diversity and stability of the intestinal microbiota, increasing the risk of disease, and even causing a decrease in the immunity of the patients, making diarrhea persistent or secondary infection. There is growing evidence that antibiotic-induced dysbiosis is associated with disease progression (Fenneman et al., 2022).

At present, there is no specific treatment for AAD in Western medicine, and its treatment is mainly through discontinuing or replacing the antibacterial drugs that induce AAD, using fecal transplantation or probiotics to improve the intestinal bacteria, but its efficacy and safety need to be further evaluated (Zhang et al., 2022). In contrast, Traditional Chinese medicine (TCM) advocates a holistic concept and the basic therapeutic concept of evidence-based treatment, and the therapeutic measures for AAD include, firstly, stopping the use of antibacterial drugs, and secondly, focusing on the fundamental treatment based on accurate evidence-based diagnosis (Shen et al., 2013). According to TCM, antibiotics are mostly bitter-cold, which can aggravate patients’ clinical symptoms with weak spleen and stomach. Therefore, the primary pathogenesis of AAD is spleen deficiency and dampness, which can be regarded as spleen deficiency diarrhea. The principle of treatment for AAD in TCM is to Jianpi Wenzhong. Qiweibaizhu powder (QWBZP) is a widely used herbal formula with good efficacy for diarrhea caused by various factors and has been commonly used in clinical practice. The complex chemical composition of QWBZP promotes the growth of beneficial intestinal bacteria. It inhibits the growth of harmful bacteria, playing an essential role in regulating the balance of microflora in the gastrointestinal tract (Liu et al., 2018). Numerous studies have shown that QWBZP can improve diarrheal disease by restoring the diversity of intestinal mucosal microbiota and regulating the structure of intestinal mucosal microbiota in mice, thereby repairing the mucosal barrier and protecting the intestine, revealing the mechanism of action of QWBZP in the treatment of AAD (Hui et al., 2020; Long et al., 2020; Sun et al., 2021). Our previous study found that QWBZP could restore the balance of the intestinal microbiota and has good efficacy in AAD. And the metabolism of Chinese herbal medicine is closely related to the intestinal microbiota. In a sense, Chinese herbal medicine treats diseases by regulating the overall environment of the human body and regulating the metabolic situation of the human intestinal mucosal microbiota (Han et al., 2020). Short-chain fatty acids (SCFAs), as one of the main metabolites of intestinal microbiota, have important roles in maintaining water-electrolyte balance, regulating intestinal microbiota balance, improving intestinal function, anti-inflammatory, anti-tumor, and regulating gene expression. Xu et al. (2020) determined the acetic acid, propionic acid, butyric acid, isobutyric acid, valeric acid, and isovaleric acid in the feces of children with spleen deficiency diarrhea and found that the levels were significantly lower than those in the feces of healthy children, suggesting the presence of intestinal microbiota dysbiosis, intestinal dysfunction, and immune imbalance of the intestinal mucosa in spleen deficiency diarrhea. Therefore, studying the changes in intestinal microbiota and SCFAs during diarrhea helps us to understand the underlying pathophysiological mechanisms.

The Chinese herbal medicines in TCM compound preparations are mainly derived from plants and animals. The secondary metabolites in plants and the special odor possessed by animal tissues make them have undesirable tastes such as bitterness, sourness, astringency, and fishy odor, thereby reducing users’ acceptability (Wu F. et al., 2022). Due to the poor taste of QWBZP, patients have difficulty taking medicine, which leads to poor compliance and limits clinical use to a certain extent. In the trend of restricting sugar intake, sweeteners have gained massive popularity. To improve the compliance of orally administered Chinese herbal medicines in clinical treatment, sweeteners are usually added to preparations to improve the taste. Sweeteners not only increase the sweetness but also mask the bitterness of the drug and adjust the taste, based on confusing the taste perception of the brain by increasing the sweetness sensation (Ayenew et al., 2009). From the perspective of TCM, sweeteners not only play a role in adjusting the taste of preparations, but some have therapeutic effects. However, it has been found that, although sweeteners have some antibacterial activity, they may also induce bacterial resistance to antibiotics by promoting the expression of bacterial efflux pumps (Yu and Guo, 2022). Sucrose is a nutritive sweetener, often used as a sweetener in preparations and for oral consumption. It has the characteristics of reasonable safety, stability, easy-to-accept taste, good water solubility, and low price. Therefore, in selecting sweeteners to improve the taste of the preparation, the dose of sweetener with the best clinical efficacy and the most negligible impact on the organism’s intestinal microbiota should be further investigated. Using intestinal microbiota as an entry point, it is vital to further investigate the effect of different sucrose dose on mice with AAD intervened by QWBZP. Therefore, this study takes the intestinal microbiota and its metabolite SCFAs as the entry point; based on the previous sweeteners research (Qiao et al., 2022), we investigated the efficacy of different sucrose dose in AAD mice intervened by QWBZP. It provided a basis for the administration of prescriptions and the selection of related excipients in the preparation development, which is essential to study the relationship between intestinal microbiota and organism health, as well as the curative effect of prescription and the addition and selection of sweeteners.

## 2. Material

### 2.1. Animals

For sex might affect intestinal microbiota (Wu Y. et al., 2022), thirty specific-pathogen-free (SPF) Kunming male mice (KM) weighing 18–22 g were purchased from Hunan Slaccas Jingda Laboratory Animal Company (Hunan, China) with license number SCXK (Xiang) 2019-0004. All animal experiments and procedures were performed according to the protocols approved by the Institutional Animal Care and Use Committee of the Hunan University of Chinese Medicine.

### 2.2. Feed

Co60 irradiated experimental rat growth and reproduction diets. The guaranteed values of the main indicators of nutrients (content per kg of feed) are shown in Table 1. The mice feeds were provided by the Experimental Animal Center of Hunan University of Chinese Medicine.

**TABLE 1:** Guaranteed values of the main indicators of the nutrient composition of Co60 irradiated experimental rat growth and reproduction diets (content per kg of feed).

Main indicators	Nutritional indicators	Main indicators	Nutritional indicators
Water content (g) ≤	100	Calcium (g)	10∼18
Crude protein (g) ≥	200	Total phosphorus (g)	6∼12
Crude fat (g) ≥	40	Calcium: Total phosphorus	1.2:1∼1.7:1
Crude fiber (g) ≥	50	Lysine (g) ≥	13.2
Crude ash (g) ≥	80	Methionine + Cystine (g) ≥	7.8

### 2.3. Medicine

According to the Chinese Pharmacopeia 2020, QWBZP was composed of *Panax ginseng* C.A.Mey., *Aucklandia lappa* Decne., *Poria cocos* (Schw.) Wolf, *Atractylodes Macrocephala* Koidz., *Pueraria lobata* (Willd.) Ohwi, *Pogostemon cablin* (Blanco) Benth, *Glycyrrhiza uralensis* Fisch. (Table 2; Qiao et al., 2022). The same batch of herbs was purchased from the First Affiliated Hospital of Hunan University of Chinese Medicine. The above is the amount of one dose of soup. Sucrose was purchased from Henan Wanbang Chemical Technology Co., Ltd.

**TABLE 2:** The ingredients of Qiweibaizhu powder (QWBZP).

Chinese name	Latin name	Place	Part used	Amount (g)	Product batch number
Renshen	*Panax ginseng* C.A.Mey.	Jilin	Root	6	SL21121302
Muxiang	*Aucklandia lappa* Decne.	Yunnan	Root	6	22030241B
Fuling	*Poria cocos* (Schw.) Wolf	Hunan	Nucleus	10	CK22052601
Baizhu	*Atractylodes Macrocephala* Koidz.	Zhejiang	Rhizome	10	2022050802
Gegen	*Pueraria lobata* (Willd.) Ohwi	Hunan	Root	10	2204130072
Huoxiang	*Pogostemon cablin* (Blanco) Benth	Guangdong	Leaves	10	CK22052402
Gancao	*Glycyrrhiza uralensis* Fisch.	Inner Mongolia	Root	3	TH22052409

### 2.4. Reagents and preparation

Preparation of mixed antibiotic solution (Shao et al., 2020): Gentamicin sulfate injection (Yichang Renfu Pharmaceutical Co., Ltd., State Drug Quantifier H42022058, product batch number: 11Y10031) and cefradine capsule (Hunan Keren Pharmaceutical Co., Ltd., State Drug Quantifier H43022214, product batch number: F220305) was prepared to make a concentration of 62.5 g/L [i.e., 6 gentamicin (2 mL/branch) + 3 cephalosporins (0.25 g/grain)] and then reserved at 4°C.

Preparation of FDA stock solution and FDA reaction solution: 2 mg of FDA is mixed with 1 mL of acetone to produce 2 mg/mL of FDA stock solution, reserved at 2-8°C and protected from light, added FDA stock solution to sterilized PBS (pH = 7.6) so that the final concentration of FDA reaction solution is 10 μg/mL (Guo et al., 2022; Li X. et al., 2022).

Preparation of phosphate buffer PBS (pH = 7.6): 4.0 g NaCl + 0.1 g KCl + 0.27 g Na_2_HPO_4_ + 0.12 g KH_2_PO_4_ was fixed to 500 mL, placed in a conical flask and sterilized before use.

Acetic acid, propionic acid, butyric acid, isobutyric acid, valeric acid, isovaleric acid, phosphoric acid, and ethyl ether were provided by Qingdao Yixin Co., Ltd.

### 2.5. Reagent kits

Mucin 2 (MUC2) and Interleukin 17 (IL-17) ELISA kits were purchased from Konotec Biotechnology Co., Ltd. DNA extraction kits: MN NucleoSpin 96 Soi, OMEGA DNA purification kits, Monarch DNA gel recovery kits were provided by Beijing Bemac Biotechnology Co., Ltd.

## 3. Method

### 3.1. Preparation of QWBZP decoction

The medicine was weighed according to the above ratio, and cold water was added to soak the surface of the medicine for 30 min. The medicine was decocted with a large flame until it boiled and then with a soft flame. The decoction time was 20–30 min. The medicine was successively decocted two times and then filtered through gauze. The liquid medicine decocted two times was mixed to make QWBZP decoction and evaporated to 0.268 g/mL in a 75°C water bath. It was found that the sugar solution mass concentration ranged from 10 to 25 g/dL for the sweetness preferred by regular people (Yang, 2006; Pu et al., 2017). Considering the *Glycyrrhiza uralensis* Fisch is a natural sweetener in QWBZP, after the sweetness test, the suitable dosage of sucrose is 8 g/dL, i.e., 8 g of sucrose per 100 mL of medicinal solution (8% sucrose). The evaporated water decoction was divided into four parts, of which three parts were added with different sucrose dose to make it into 4% sucrose plus QWBZP, 8% sucrose plus QWBZP, 12% sucrose plus QWBZP. The configured solution was reserved at 4°C and rewarmed to 25–30°C before use.

### 3.2. Animal groups

**FIGURE 1:**
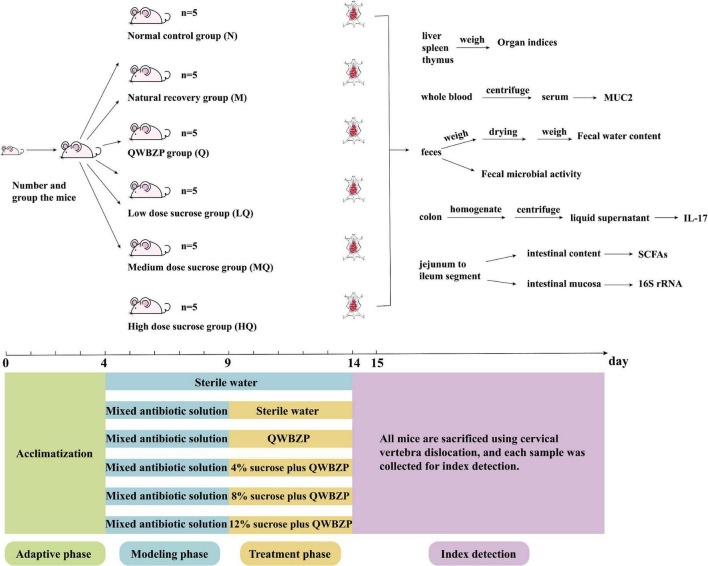
Experimental flow chart.

After 4 days of adaptive rearing in a suitable environment (room temperature 23–25°C, relative humidity 50–70%, clean and quiet), Thirty KM mice were randomly divided into normal group (N), natural recovery group (M), QWBZP group (Q), low dose sucrose group (LQ), medium dose sucrose group (MQ), and high dose sucrose group (HQ), with five mice in each group, and the mice were numbered and reared in separate cages (Figure 1).

### 3.3. Modeling and treatment

According to reference (Guo et al., 2019), mice in the M, Q, LQ, MQ, and HQ groups were administered an antibiotic mixture of gentamicin sulfate and cephradine, 23.33 mL/(kg d) two times per day for 5 days. Correspondingly, mice in the N group were gavaged with sterile water. When diarrhea symptoms were induced (declined activity, arched back trembling, specifically watery stool, curled up, and poor appetite), mice in the Q, LQ, MQ, and HQ groups were gavaged with QWBZP, 4% sucrose plus 7.15 g/(kg⋅d) QWBZP, 8% sucrose plus 7.15 g/(kg⋅d) QWBZP, and 12% sucrose plus 7.15 g/(kg⋅d) QWBZP, respectively, two times per day for 5 days. Correspondingly, mice in the N and M groups were gavaged with sterile water (Figure 1).

### 3.4. General features observation

The general status of the mice in each group was observed during the adaptive phase, modeling phase, and treatment phase, including body weight, food intake, activity status, mental status, stool pattern, and the presence of rectocele, etc.

### 3.5. Weight gain rate determination

The mice were weighed before the operation on the 2nd day of adaptive phase, at the end of adaptive phase (day 1 of modeling), at the end of modeling (day 1 of treatment), and at the end of treatment (day of sampling), respectively. The formula for calculating the body weight change rate was as follows:

End of adaptive phase weight gain rate = (body weight at the end of adaptive phase − body weight on the 2nd day of adaptive phase)/body weight on the 2nd day of adaptive phase × 100%

End-of-modeling weight gain rate = (end-of-modeling body weight − end-of-adaptation body weight)/end-of-adaptation body weight × 100%

End-of-treatment weight gain rate = (end-of-treatment body weight − end-of-modeling body weight)/end-of-modeling body weight × 100%

### 3.6. Fecal water content determination

Fresh fecal samples were collected from each group of mice before the operation (e.g., before gavage) from the first day of modeling to the last day of treatment, and recorded the wet weight of each fecal sample was. Then dried the fecal samples to constant weight, and record the dry weight of each stool sample was to calculate the fecal moisture content: fecal water content (%) = (wet weight of feces − dry weight of feces)/wet weight of feces × 100%

### 3.7. Fecal microbial activity determination

Equal-weight fecal samples were weighed for each group, and put into a sterilized centrifugal tube containing glass beads. Blank control group: Added 2 mL FDA reaction solution, 2 mL acetone, and 50 μL sample into a dry sterilized tube, and the mixture was incubated at 24°C for 90 min. Experimental group: Added 2 mL FDA reaction solution and 50 μL sample into a dry sterilized tube, and the mixture was incubated at 24°C for 90 min, subsequently removed, and added 2 mL acetone was to terminate the reaction. Finally, all samples were determined three times in parallel by UV spectrophotometer at 490 nm, and the microbial activity per unit mass of the sample is expressed as A_490_ value (Guo et al., 2022; Li X. et al., 2022).

### 3.8. MUC2 content and IL-17 content determination

According to the kit instructions, the MUC2 content of serum samples and the IL-17 content of colon samples were detected by enzyme-linked immunosorbent assay.

### 3.9. Organ indices determination

The body weight of each mouse was weighed before sampling. After the mice were sacrificed using cervical vertebra dislocation on a sterile operation platform, the intact liver, spleen, and thymus were removed, and the attached surface fascia and the adipose tissue were also removed. Then, blood was blotted from the organ surface with filter paper and weighed separately, and the liver, spleen, and thymus indices were calculated: organ index = organ weight (mg)/body weight (g).

### 3.10. Intestinal contents samples and intestinal mucosa samples extraction

After the mice were sacrificed using cervical vertebra dislocation on a sterile operation platform, the jejunum to ileum segment was taken as the test specimens. Squeezed out the contents of the chymus, individually loaded into 1.5 mL sterilized ep tubes numbered and then quickly frozen in liquid nitrogen, and finally reserved at −80°C for short-chain fatty acid determination. Subsequently, the intestine was dissected, and the intestinal wall was washed with saline. The intestinal wall tissues were retrieved and blotted with filter paper to absorb excess water and then scraped with coverslips, individually loaded into 1.5 mL sterilized ep tubes numbered and then quickly frozen in liquid nitrogen, and finally reserved at −80°C for sequencing.

### 3.11. SCFAs determination

The qualitative analysis of SCFAs was entrusted to Qingdao Yixin Co., Ltd. Preparation of standards: take about 0.1 g of acetic acid, propionic acid, butyric acid, isobutyric acid, valeric acid, isovaleric acid with 100 mL volumetric flask, add ether to fix the volume as reserve solution; take 1, 0.75, 0.5, and 0.25 mL reserve solution with 100 mL volumetric flask, fix the volume with ether, configure well and feed the sample for analysis. Sample pretreatment: take the sample and add 2 mL water (1:3 phosphoric acid aqueous solution), vortex homogenization for 2 min, add 1 mL ether extraction for 10 min, and put it in the ice water bath with 4,000 r/min low-temperature centrifugation for 20 min, then add 1 mL ether extraction for 10 min 4,000 r/min centrifugal separations; combine the two extractions and volatilize to within 1 mL, and feed the sample for analysis. Thermo Fisher GCMS ISQ LT gas chromatograph was used. Temperature rise program setting: 100°C (5 min) → 5°C/min → 150°C (0 min) → 30°C/min → 240°C (30 min); flow rate: 1 mL/min; split ratio: 75:1; carrier gas: helium; column: TG WAX 30 m × 0.25 mm × 0.25 μm; injector: 240°C; mass spectrometer: EI source; bombardment voltage: 70 eV; single ion scan mode: quantitative ion 62, 73; ion source temperature: 200°C; connection line temperature: 250°C.

### 3.12. Intestinal mucosal microbiota determination

The sample DNA extraction, amplification, and library sequencing were provided by Beijing Bemac Biotechnology Co., Ltd.

#### 3.12.1. DNA extraction and amplification

Total DNA was extracted from the intestinal mucosa samples of each group of mice, and the extraction method was performed according to the DNA extraction kit (MN NucleoSpin 96 Soi). The concentration of the extracted DNA was determined using Nanodrop, and the purity of the extracted DNA was checked by 1.8% agarose gel electrophoresis. The V3 + V4 extremely variable region of the 16S rRNA gene was selected as the PCR amplification region, and the qualified DNA was amplified using 16S rRNA universal primers (338F/806R): upstream primer 338F (5′-ACTCCTACGGGAGGCAGCA-3′) and downstream primer 806R (5′-GGACTACHVGGGTWTCTAAT-3′). The amplification 10 μL system included: Vn F (10 μmol/L) 0.3 μL, Vn R (10 μmol/L) 0.3 μL, KOD FX Neo Buffer 5 μL, dNTP (2 mmol/L each) 2 μL, KOD FX Neo 0.2 μL, and ddH_2_O was added to 10 μL. Amplification reaction conditions: 95°C pre-denaturation for 5 min, followed by 25 cycles: denaturation at 95°C for 30 s, annealing at 50°C for 30 s, extension at 72°C for 40 s, and final extension at 72°C for 7 min, and termination at 4°C.

#### 3.12.2. Purification recovery and quantification of amplification products

PCR products were mixed at a mass ratio of 1:1 according to the results of electrophoresis quantification (ImageJ software). After mixing, the product was purified by OMEGA DNA purification kit. 1.8% agarose gel, 120 V for 40 min electrophoresis was used to cut the target fragment and recovered by Monarch DNA gel recovery kit.

## 4. Bioinformatic and statistical analysis

Using USEARCH (version 10.0) software platform, sequences were clustered at the 97% level of similarity, and operational taxonomic units (OTU) was filtered by default using 0.005% of the number of all lines sequenced as the threshold. Dilution curve, Shannon–Wiener curve, and Coverage index were used to evaluate the sequencing depth of 16S rRNA gene of intestinal microbiota. The alpha and beta diversities were demonstrated by Chao1, Shannon, ACE, Simpson index, principal co-ordinates analysis (PCoA), non-metric multi-dimensional scaling (NMDS) analysis, and analysis of similarities (ANOSIM). The abundance of microbiota at all levels is calculated based on the OTU and is presented in a histogram. Our study uses line discriminant analysis effect size (LEfSe) to find the biomarker for different groups. To evaluate the diagnostic efficiency of the differential genera selected by LEfSe analysis, the ROC curve of each statistically significant differential genus was constructed, and the area under the curve (AUC) was calculated. The AUC is the area under the ROC enclosed by the coordinate axis. AUC usually ranges from 0 to 1. The closer the AUC is to 1, the more it is considered to be the flora with relative abundance difference and diagnostic efficacy in both groups. To further analyze the effect of different sucrose dose on the correlation between intestinal mucosal microbiota and environmental factors in AAD mice intervened by QWBZP, we calculated the Spearman correlation coefficient and plotted a correlation heat map. In addition, we classified the Kyoto Encyclopedia of Genes and Genomes (KEGG) using PICRUSt2.

Statistical analysis was performed using SPSS 25.00 software, and the data obtained for each group was expressed as mean ± standard deviation (mean ± SD). When the data conformed to normal distribution, the independent sample *t*-test was used for comparative analysis between two groups; one-way ANOVA was used for comparative analysis between multiple groups, and LSD method was used for two-way comparison between groups. Otherwise, Mann–Whitney *U* test was used for comparison between two groups, and Kruskal–Wallis *H* test was used for comparison between multiple groups. The test level was set at α = 0.05. When *p* < 0.05, the result statistics were significantly different; when *p* < 0.01, the result statistics were extremely significant difference.

## 5. Results and analysis

### 5.1. General features observation

#### 5.1.1. The general physical signs of mice in the adaptive, modeling, and treatment phases

During the adaptive phase, the mice had regular food intake, sensitive reactions, and smooth and shiny hair. During the modeling phase, mice showed a clustering phenomenon, decreased food intake, increased water intake, increased urine output, and tarnished and disordered hair. However, no significant abnormality was observed in the mice of the N group. During the treatment phase, the water intake and urine output of mice in the N group increased. In the M group, the food intake was decreased, the water intake was increased, and the urine output was increased. The mice liked to curl up and had dry hair. The mice in each treatment group showed flexible activity, and the hair recovered from being lustrous. The food intake, water intake, and urine output were all restored to normal, and there was a dose-response relationship between food intake and sucrose dose, suggesting that QWBZP and QWBZP with different sucrose dose had a certain effect on improving the food intake, water intake and other related symptoms in mice with AAD.

#### 5.1.2. The fecal characteristics of mice in the adaptive, modeling, and treatment phases

**FIGURE 2:**
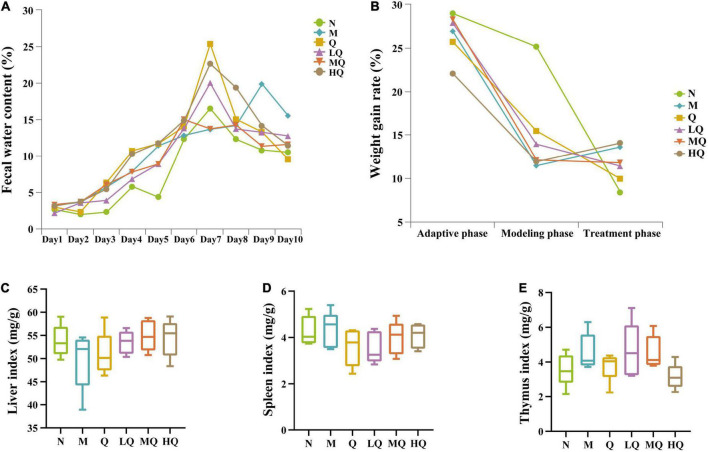
Effect of antibiotic modeling on normal mice and different sucrose dose on mice with antibiotic-associated diarrhea (AAD) intervened by Qiweibaizhu powder (QWBZP). **(A)** Fecal water content; **(B)** Weight gain rate; **(C)** Liver index; **(D)** Spleen index; **(E)** Thymus index.

During the adaptive phase, the feces of mice were black, granular, dry, and non-sticky after being crushed. As shown in Figure 2A, the mice developed diarrhea one after another from the 2nd to the 5th day after modeling, and prominent diarrhea appeared in all groups on the 5th day. The clinical manifestations were thin and soft feces with yellow watery stool and rectocele in some mice. However, no significant abnormality was observed in the mice of the N group, indicating that the AAD model was successfully established. Subsequently, the mice were treated with QWBZP and QWBZP with different sucrose dose. From the first to the second day of treatment, the diarrhea of the mice in each treatment group remained unchanged, and the feces were wet, speculated as the delayed response of antibiotic modeling. From the third to the fifth day of treatment, compared with the mice in the N group, the feces of the mice in the M group were still loose and soft, and the feces were granular, and they became sticky after being pressed. The mice in each treatment group gradually stopped diarrhea, and the feces were granular, neither dry nor wet, suggesting that the treatment with QWBZP and QWBZP with different sucrose dose had certain efficacy in mice with AAD. The increase in the fecal water content of the mice in the N group during the treatment phase was presumably due to the increased water intake of the mice.

### 5.2. Effect of different sucrose dose on the weight gain rate of mice with AAD intervened by QWBZP

As can be seen from Figure 2B, the weight gain rate was comparable in all groups of mice during the adaptive phase. After the gavage of mixed antibiotic solution, the weight gain rate of mice decreased significantly, and all of them were significant difference from the N group (*p* < 0.05), which showed that the gavage of mixed antibiotics affected the food intake amount of mice. After the administration of drug treatment, there was no significant change in the weight gain rate of mice in each group, and there was no significant difference (*p* > 0.05). Combined with the observation of the general state of mice, it was presumed that each treatment group had a certain effect on restoring the food intake of mice and alleviating diarrhea. Still, there was no absolute significance of the weight gain rate.

### 5.3. Effect of different sucrose dose on the organ indices of mice with AAD intervened by QWBZP

As can be seen from Figures 2C–E, compared with the N group, the liver index decreased in the M group, while the liver index in the Q, LQ, MQ, and HQ groups were similar to the N group, but there was no significant difference (*p* > 0.05). The spleen and thymus indices in each group also showed no significant difference (*p* > 0.05).

### 5.4. Effect of different sucrose dose on the fecal microbial activity of mice with AAD intervened by QWBZP

**FIGURE 3:**
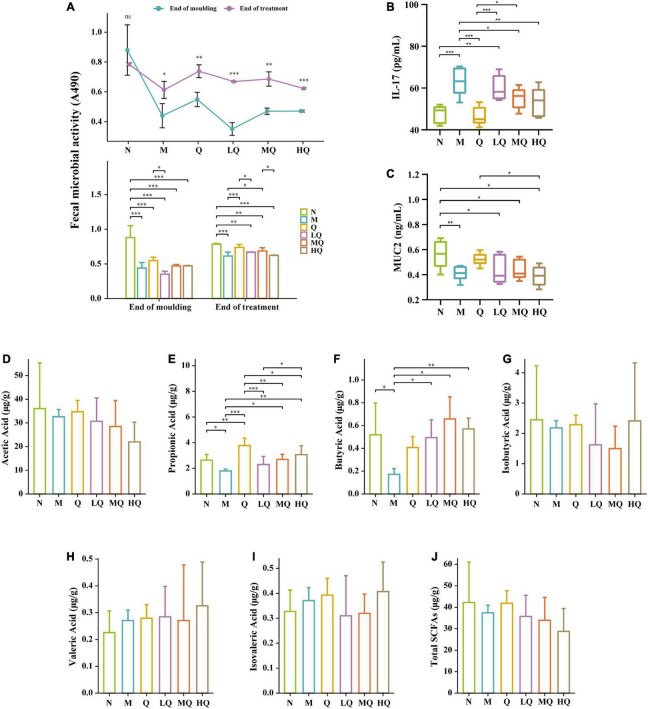
Effect of different sucrose dose on the fecal microbial activity **(A)**, IL-17 content **(B)**, mucin 2 (MUC2) content **(C)**, and short-chain fatty acids (SCFAs) **(D–J)** of mice with antibiotic-associated diarrhea (AAD) intervened by Qiweibaizhu powder (QWBZP). *p < 0.05; **p ≤ 0.01; ***p ≤ 0.001.

As shown in Figure 3A, compared with the N group, the antibiotic modeling caused an extremely significant decrease in the fecal microbial activity of AAD mice (*p* = 0.000). After 5 d of treatment, fecal microbial activity was significantly increased in all treatment groups (*p* < 0.05). Compared with the M group, the fecal microbial activity was significantly increased in the Q and MQ groups (*p* < 0.05). It also increased in the LQ and HQ groups, but there was no significant difference (*p* > 0.05).

### 5.5. Effect of different sucrose dose on the content of inflammatory factor IL-17 and intestinal mucosal protective factor MUC2 in AAD mice intervened by QWBZP

As shown in Figure 3B, the IL-17 content was increased extremely significantly in the M group (*p* ≤ 0.001). Compared with the M group, the IL-17 content was decreased in each treatment group, and the IL-17 content from low to high was ranked as Q group (*p* ≤ 0.001), HQ group (*p* ≤ 0.01), MQ group (*p* < 0.05), and LQ group (*p* > 0.05).

As shown in Figure 3C, the MUC2 content was significantly decreased in the M group (*p* < 0.05). Compared with the M group, the MUC2 content was increased in the Q, LQ, and MQ groups, and the MUC2 content from low to high was ranked as LQ, MQ, and Q groups, but there was no significant difference (*p* > 0.05). In contrast, the MUC2 content in the HQ group continued to decrease, but there was no significant difference too (*p* > 0.05).

### 5.6. Effect of different sucrose dose on the SCFAs of mice with AAD intervened by QWBZP

As shown in Figures 3D–J, the contents of acetic acid, propionic acid (*p* < 0.05), butyric acid (*p* < 0.05), and isobutyric acid (*p* > 0.05) in the intestinal contents decreased in the M group. Compared with the M group, the content of all six SCFAs increased in the Q group, with an extremely significant increase in propionic acid content (*p* ≤ 0.001). In the LQ, MQ, and HQ groups, the content of acetic acid showed a decreasing trend, but there was no significant difference (*p* > 0.05); the content of propionic acid was all significantly lower than that of the Q group (*p* ≤ 0.001, *p* ≤ 0.01, *p* < 0.05); the content of butyric acid was all significantly higher than that of the M group (*p* < 0.05, *p* ≤ 0.01, *p* ≤ 0.001).

### 5.7. Effect of different sucrose dose on the intestinal mucosal microbiota of mice with AAD intervened by QWBZP

#### 5.7.1. Effect of different sucrose dose on the alpha diversity of mice with AAD intervened by QWBZP

**FIGURE 4:**
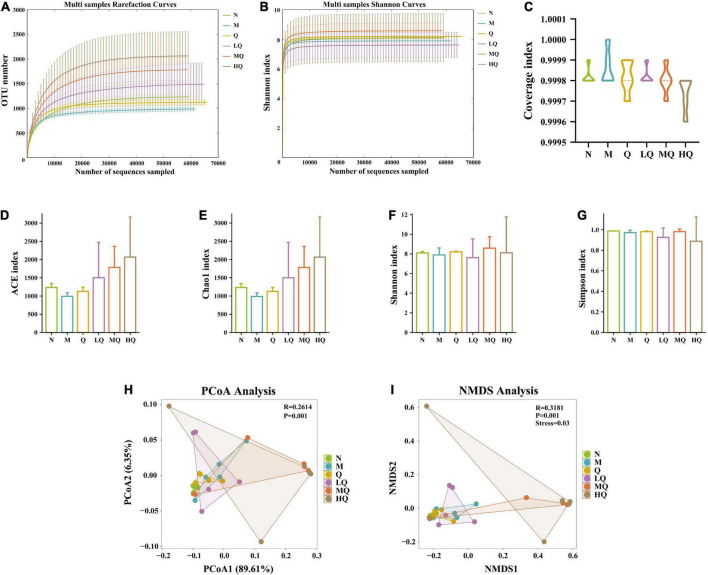
Effect of different sucrose dose on the alpha diversity and beta diversity of mice with antibiotic-associated diarrhea (AAD) intervened by Qiweibaizhu powder (QWBZP). **(A)** Rarefaction curve; **(B)** Shannon–Wiener curve; **(C)** Coverage index; **(D)** ACE index; **(E)** Chao1 index; **(F)** Simpson index; **(G)** Shannon index; **(H)** Principal co-ordinates analysis (PCoA) analysis; **(I)** Non-metric multi-dimensional scaling (NMDS) analysis.

The rarefaction curves are constructed by random sampling of sequences, using the number of sequences drawn versus the number of OTUs they can represent, and can be used to compare the richness of species in samples with different amounts of sequencing data and to indicate whether the amount of sequencing data in a sample is reasonable. The Shannon–Wiener curve was constructed using the microbial diversity index at different sequencing depths for each sample to reflect the microbial diversity of each sample at different sequencing amounts. In this experiment, the rarefaction curve and Shannon–Wiener curve both tended to be flat, indicating that the sequencing data were reasonable and the sequencing depth was reliable enough to reflect the majority of microbial information in the samples (Figures 4A, B). The values of the coverage index were all greater than 0.999, indicating that the sequences in the samples were detected basically (Figure 4C).

Alpha diversity describes the biodiversity of a particular region or ecosystem, i.e., the assessment of the biodiversity of a sample, and is generally characterized by a diversity index based on species richness or evenness. The Chao1 and ACE indices are commonly used to estimate the total number of community species, with larger indices indicating a higher total number of community species. The Shannon and Simpson indices can objectively reflect the diversity of community species. The smaller the value of the Simpson index, the higher the diversity of the community; the larger the value of the Shannon index, the higher the community diversity. The results in Figures 4D–G showed that the diversity indices of intestinal mucosal samples were changed to some extent. Still, there was no significant difference, among which the diversity indices of the Q group always tended to the N group.

#### 5.7.2. Effect of different sucrose dose on the beta diversity of mice with AAD intervened by QWBZP

Beta diversity describes the differences in species composition between different habitat communities, i.e., comparing differences between samples. As shown in Figures 4H, I, the differences in microbial community composition between groups were extremely significant (*p* = 0.001), which was also confirmed by Anosim. PCoA analysis showed (Figure 4H) that there was no overlap between the N group and the M group, indicating that antibiotic modeling affected the structure of the intestinal mucosal microbiota. The Q group and the LQ group had less overlap with the M group and were close to the N group in terms of distance, i.e., these two groups were less similar to the M group and more similar to the N group. It was presumed that the Q and LQ groups had a better effect on restoring intestinal mucosal microbiota structure. The MQ group overlapped highly with the M group, and the HQ group did not overlap with the M group but was far away from the N group in terms of distance. It was presumed that the restoration of intestinal mucosal microbiota structure was poor with the MQ and HQ groups. NMDS analysis (Figure 4I) was consistent with the results of PCoA analysis.

#### 5.7.3. Effect of different sucrose dose on the OTUs number of mice with AAD intervened by QWBZP

**FIGURE 5:**
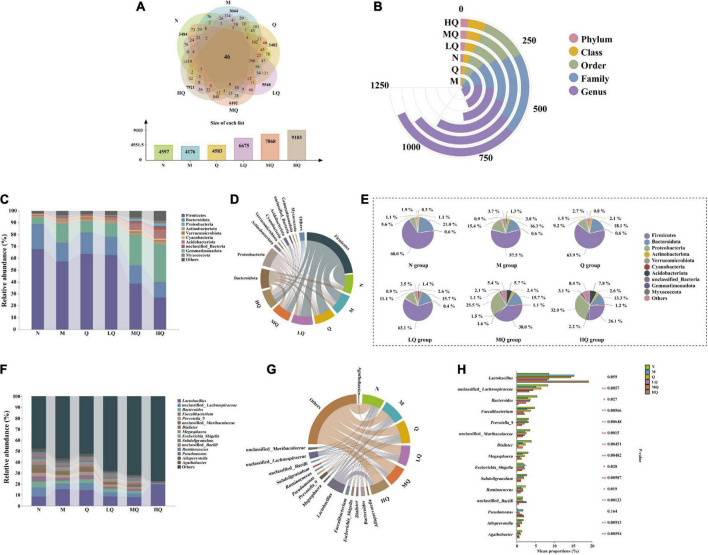
Effect of different sucrose dose on the intestinal mucosal microbiota structure of mice with antibiotic-associated diarrhea (AAD) intervened by Qiweibaizhu powder (QWBZP). **(A)** Operational taxonomic units (OTUs) number; **(B)** Interactive Yujue diagram; **(C)** Histogram of relative abundance at the phylum level; **(D)** Chord diagram of the phylum level; **(E)** Pie chart of the phylum level; **(F)** Histogram of relative abundance at the genus level; **(G)** Chord diagram of the genus level; **(H)** Multi-species comparison at the genus level.

Operational taxonomic unit (OTU) Venn diagrams analyze the similarity and overlap of community structures of different samples and visualize the similarity and uniqueness of samples at the OTU level. Using USEARCH (version 10.0) software platform, sequences were clustered at the 97% level of similarity, and OTUs were filtered by default using 0.005% of the number of all lines sequenced as the threshold (Figure 5A). The total number of OTUs for the six experimental groups was 46. There were 4,597, 4,176, and 4,583 OTUs found in the N, M, and Q groups, respectively, which showed that antibiotic modeling reduced the total number of OTUs in the intestinal mucosal microbiota and the number of unique OTUs decreased accordingly. After treatment with QWBZP, it recovered to the normal level. There were 6,675, 7,868, and 9,103 OTUs found in the LQ, MQ, and HQ groups, respectively, which indicated that sucrose intake significantly changed the intestinal microbial species and increased the diversity of intestinal mucosal microbiota.

#### 5.7.4. Effect of different sucrose dose on the relative abundance of intestinal mucosal microbiota (at phylum level and genus level) of mice with AAD intervened by QWBZP

By comparing the changes in the composition of each taxonomic level in each group of mice (Figure 5B), it was found that the addition of sucrose could significantly change the content of mice intestinal contents microbiota in five taxonomic levels (phylum, class, order, family, and genus).

Figure 5C shows the relative abundance of the top 10 bacteria at the phylum level. Among them, Firmicutes, Bacteroidota, Proteobacteria, Actinobacteriota, Acidobacteriota, and Verrucomicrobiota are the dominant phylum with a large proportion of the six groups (Figure 5D). As shown in Figure 5E, after antibiotic modeling, the relative abundance of Firmicutes, Bacteroidota, and Verrucomicrobiota decreased by 10.5, 4.7, and 0.4%. At the same time, that of Proteobacteria, Actinobacteriota, and Acidobacteriota increased by 9.8, 1.9, and 0.9%, but none of them were significant differences. After treatment with QWBZP, the relative abundance of Firmicutes, Bacteroidota, and Verrucomicrobiota increased by 6.4, 1.8, and 0.8%. At the same time, that of Proteobacteria, Actinobacteriota, and Acidobacteriota decreased by 6.2, 0.2, and 0.8%, but none of them were significant differences. With increasing sucrose dose, the relative abundance of Firmicutes, Bacteroidota, and Proteobacteria tended to decrease. In contrast, that of Actinobacteriota, Acidobacteriota, and Verrucomicrobiota tended to increase, but none of them were significant difference.

Figures 5F, G shows that there are 15 main dominant genera in the intestinal mucosal microbiota. As can be seen from Figure 5H, among them, unclassified_*Lachnospiraceae*, *Bacteroides*, *Faecalibacterium*, *Prevotella*_9, unclassified_*Muribaculaceae*, *Dialister*, *Megasphaera*, *Escherichia_Shigella*, *Subdoligranulum*, unclassified_*Bacilli*, *Ruminococcus*, *Alloprevotella*, and *Agathobacter*, 13 genera with significant differences. Antibiotic modeling upregulated the relative abundance of *Escherichia_Shigella*. In contrast, unclassified_*Lachnospiraceae*, *Bacteroides*, *Faecalibacterium*, *Prevotella*_9, unclassified_*Muribaculaceae*, *Dialister*, *Megasphaera*, *Subdoligranulum*, unclassified_*Bacilli*, *Ruminococcus*, *Alloprevotella*, and *Agathobacter* were significantly downregulated. After treatment with QWBZP, the relative abundance of the above genera showed an opposite trend. Compared with the M group, the relative abundance of unclassified_*Lachnospiraceae*, *Faecalibacterium*, *Prevotella*_9, *Megasphaera*, *Subdoligranulum*, *Alloprevotella*, and *Agathobacter* were reduced in the LQ, MQ, and HQ groups.

#### 5.7.5. Effect of different sucrose dose on the characteristics of intestinal mucosal microbiota in mice with AAD intervened by QWBZP

**FIGURE 6:**
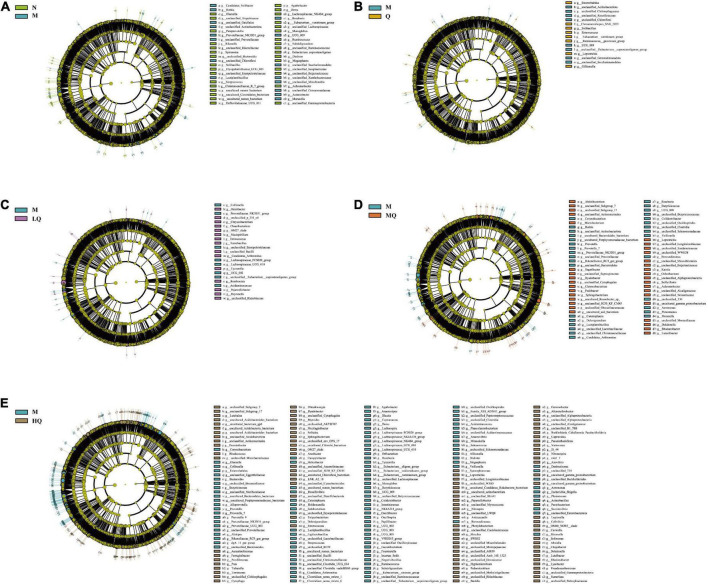
Effect of different sucrose dose on the characteristic of intestinal mucosal microbiota in antibiotic-associated diarrhea (AAD) mice intervened by Qiweibaizhu powder (QWBZP). **(A)** N group and M group; **(B)** M group and Q group; **(C)** M group and LQ group; **(D)** M group and MQ group; **(E)** M group and HQ group.

We used LEfSe analysis to identify microbiota that was significantly different between groups. The characteristic genera between the N and M groups are shown in Figure 6A: unclassified_ *Lachnospiraceae*, *Dialister*, *Megasphaera*, etc. were the characteristic genera enriched in the N group; *Moraxella*, unclassified_ *Prevotellaceae*, *Rothia*, etc. were the characteristic genera enriched in the M group. The characteristic genera between the Q and M groups are shown in Figure 6B: *Enterococcus*, *Solibacillus*, [*Eubacterium*]_ *ventriosum*_group, etc. were the characteristic genera enriched in the Q group; unclassified_[*Eubacterium*]_*coprostanoligenes*_group, unclassified_*Chloroflexi*, unclassified_*Roseiflexaceae*, etc. were the characteristic genera enriched in the M group. The characteristic genera between the LQ and M groups are shown in Figure 6C: *Candidatus_Arthromitus*, unclassified_*Bacilli*, *Deinococcus*, etc. were the characteristic genera enriched in the LQ group; UCG_ 002, *Prevotellaceae*_NK3B31_group, *Collinsella*, etc. were the characteristic genera enriched in the M group. The characteristic genera between the MQ and M groups are shown in Figure 6D: unclassified_g_Subgroup_17, *Rhodanobacter*, uncultured_gamma_ *proteobacterium*, etc. were the characteristic genera enriched in the MQ group; *Brevundimonas*, *Candidatus_Arthromitus*, *Moraxella*, etc. were the characteristic genera enriched in the M group. The characteristic genera between the HQ and M groups are shown in Figure 6E: *Syntrophomonas*, *Myroides*, *Nitrospira*, etc. were the characteristic genera enriched in the HQ group; unclassified_ *Lachnospiraceae*, *Brevundimonas*, *Prevotella*_9, etc. were the characteristic genera enriched in the M group.

#### 5.7.6. Effect of different sucrose dose on the functional analysis of the intestinal mucosal microbiota in mice with AAD intervened by QWBZP

**FIGURE 7:**
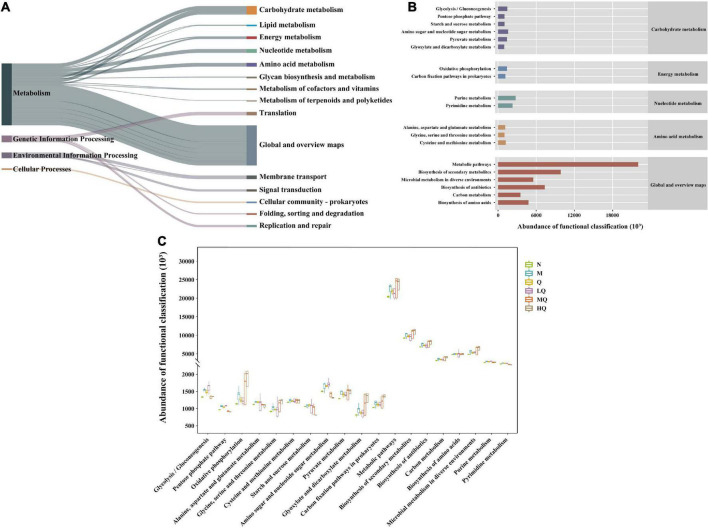
PICRUst-based examination of intestinal mucosal microbiota; **(A)** Kyoto Encyclopedia of Genes and Genomes (KEGG) functional categories (levels 1 and 2). **(B)** Metabolic histogram (levels 2 and 3). **(C)** Comparisons between the groups for each KEGG functional categories (level 3).

The intestinal mucosal microbiota function was generally divided into six categories, and the second level included 46 sub-functional categories, of which the median value > 711428.9, with 15 categories (Figure 7A). The abundance of metabolic functions was higher, with 152 categories, of which the median value > 979661.4, with 19 categories (Figure 7B). As shown in Figure 7B, it has a great influence on global and general overview maps, carbohydrate metabolism, and amino acid metabolism. Further analysis revealed that there were significant differences in glycolysis/gluconeogenesis, pentose phosphate pathway, purine metabolism, pyrimidine metabolism, starch, and sucrose metabolism (Figure 7C).

#### 5.7.7. Correlation analysis between characteristic genera, IL-17, MUC2, and SCFAs

To further analyze the effect of different sucrose dose on the correlation between intestinal mucosal microbiota and environmental factors in AAD mice intervened by QWBZP, we used Spearman’s method to screen the top five characteristic genera in each group for correlation analysis with inflammatory factor IL-17, intestinal mucosal protective factor MUC2, and metabolites SCFAs, respectively. To verify the predictive accuracy of the screened characteristic genera, we used AUC > 0.8 as a criterion to verify the accuracy of different intergroup diagnoses and joint evaluation by characteristic bacteria in each group and to determine whether they had diagnostic efficacy. The characteristic bacteria with AUC > 0.8 is defined as an important bacteria that describes the different characteristics between the two groups.

**FIGURE 8:**
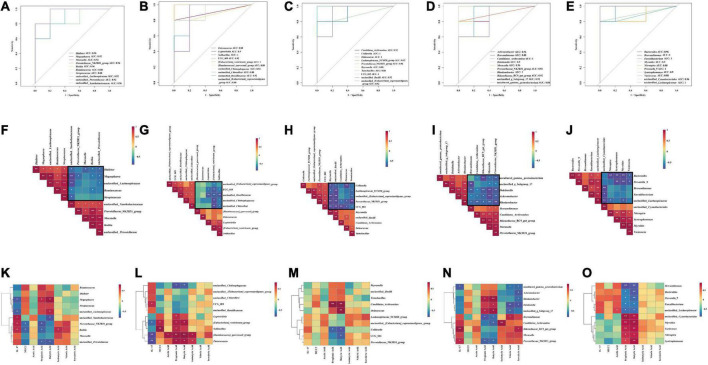
ROC curve analysis: **(A)** N group and M group; **(B)** M group and Q group; **(C)** M group and LQ group; **(D)** M group and MQ group; **(E)** M group and HQ group; Correlation analysis between characteristic genera: **(F)** N group and M group; **(G)** M group and Q group; **(H)** M group and LQ group; **(I)** M group and MQ group; **(J)** M group and HQ group; Correlation analysis between characteristic genera, IL-17, MUC2 and SCFAs: **(K)** N group and M group; **(L)** M group and Q group; **(M)** M group and LQ group; **(N)** M group and MQ group; **(O)** M group and HQ group. The legend shows the correlation coefficient values. Red represents positive correlation, blue represents negative correlation, and the gradient of color indicates the strength of correlation. *p < 0.05 and **p < 0.01.

From the ROC plot (Figures 8A–E), it was found that all the characteristic genera screened in each group had AUC > 0.8, which can be considered as the bacteria with diagnostic efficacy.

As shown in Figure 8F, Spearman correlation analysis of the characteristic genera of the two groups of N and M groups showed a significant association between the characteristic genera. As shown in the black box, there was a negative correlation trend, and it was speculated that antibiotic modeling changed the structure of the intestinal microbiota.

As shown in Figures 8G–J, Spearman’s correlation analysis of the characteristic genera of the M and Q groups, the M and LQ groups, the M and MQ groups, and the M and HQ groups, respectively, showed a significant association between the characteristic genera. As shown in the black box, all showed a negative correlation trend, and it was speculated that the treatment of QWBZP, low dose sucrose plus QWBZP, medium dose sucrose plus QWBZP, and high dose sucrose plus QWBZP changed the microbiota structure of the AAD mice.

Compared to the N and M groups, the association heat map revealed that *Megasphaera* showed a significant negative correlation with IL-17 and a significant positive correlation with propionic acid and butyric acid; unclassified_*Lachnospiraceae* showed a significant negative correlation with IL-17 and a significant positive correlation with butyric acid; all of the above are the characteristic genera of the N group. unclassified_*Xanthobacteraceae* showed a significant positive correlation with IL-17 and a significant negative correlation with butyric acid; unclassified_*Prevotellaceae* showed a significant negative correlation with propionic acid and butyric acid; *Prevotellaceae*_NK3B31_group showed a significant positive correlation with IL-17 and a significant negative correlation with MUC2 and propionic acid; *Rothia* showed a significant negative correlation with IL-17, isobutyric acid and a significant negative correlation with MUC2, propionic acid; all of the above were characteristic genera of the M group (Figure 8K).

Compared to the M and Q groups, the association heat map revealed that unclassified_*Chitinophagaceae* showed a significant negative correlation with propionic acid and butyric acid; UCG_009 showed a significant positive correlation with IL-17; all of the above were characteristic genera of the M group. *Leptotrichia* showed a significant positive correlation with propionic acid; [*Eubacterium*]_*ventriosum*_group showed a significant negative correlation with IL-17 and a significant positive correlation with MUC2, propionic acid, and butyric acid; *Solibacillus* showed a significant positive correlation with MUC2 and propionic acid; [*Ruminococcus*]_*gauvreauii*_group showed a significant negative correlation with IL-17 and a significant positive correlation with acetic acid, propionic acid, butyric acid, and isobutyric acid; *Enterococcus* showed a significant negative correlation with IL-17 and a significant positive correlation with propionic acid, butyric acid, and isobutyric acid; all of the above were the characteristic genera of the Q group (Figure 8L).

Compared to the M and LQ group, the association heat map revealed that *Collinsella* showed a significant negative correlation with propionic acid and butyric acid; UCG_002 showed a significant negative correlation with butyric acid; all of the above were the characteristic genera of the M group; *Candidatus_Arthromitus* showed a significant positive correlation with propionic acid and butyric acid; *Deinococcus* showed a significant positive correlation with butyric acid; all of the above were the characteristic genera of the LQ group (Figure 8M).

Compared to the M and MQ groups, the association heat map revealed that *Brevundimonas* showed a significant negative correlation with propionic acid, butyric acid, and a significant positive correlation with valeric acid; *Rikenellaceae*_RC9_gut_group showed a significant positive correlation with IL-17 and isovaleric acid; *Candidatus_ Arthromitus* showed a significant negative correlation with butyric acid and a significant positive correction with isobutyric acid; *Prevotellaceae*_NK3B31_group showed a significant positive correlation with IL-17 and a significant negative correlation with propionic acid and butyric acid; all of the above were characteristic genera of the M group. uncultured_gamma_*proteobacterium* showed a significant negative correlation with IL-17, valeric acid, and isovaleric acid; *Dokdonella* showed a significant positive correlation with butyric acid and a significant negative correlation with valeric acid and isovaleric acid; unclassified_g_Subgroup_17 showed a significant positive correlation with propionic acid, butyric acid and a significant negative correlation with valeric acid. *Achromobacter* showed a significant negative correlation with isovaleric acid; *Rhodanobacter* showed a significant positive correlation with propionic acid, butyric acid and a significant negative correlation with isovaleric acid; all of the above were the characteristic genera of the MQ group (Figure 8N).

Compared to the M and HQ groups, the association heat map revealed that *Brevundimonas*, *Prevotella*_9, *Faecalibacterium*, and *Bacteroides* showed a significant negative correlation with propionic acid and butyric acid; unclassified_*Lachnospiraceae* showed a significant positive correlation with acetic acid and a significant negative correlation with propionic acid and butyric acid; all of the above were characteristic genera of the M group. unclassified_*Cyanobacteriales*, *Syntrophomonas*, *Myroides*, *Nitrospira*, and *Variovorax* showed a significant positive correlation with propionic acid and butyric acid; all of the above were characteristic genera of the HQ group (Figure 8O).

## 6. Discussion

Natural sweeteners (sucrose, etc.) are widely used in the food industry in China and worldwide. After ingestion of sweet substances such as natural sweeteners or artificial sweeteners, the taste information of animals is perceived by their sweet taste receptors (Liu and Bohórquez, 2022), and corresponding chemical signals are generated on the taste buds and transmitted to the central nervous system, causing the release of regulatory factors associated with feeding. However, when the dose of sweetness is too high, it will produce a negative feedback regulator for animals, suppressing their appetite and reducing feed intake. Studies have shown that chronic exposure to sucrose and acesulfame solutions affects behavioral indicators such as food intake, body weight, and neurotransmitter release in mice (Yin et al., 2020). It was found that body weight change is a complex process, and a single factor does not directly influence it. Due to differences in body function, intestinal microbiota, gender, etc., mice ingested sweeteners did not necessarily result in increased body weight (Bian et al., 2017; Yunker et al., 2021). In this experiment, it was observed that the mice gradually returned to regular food intake after treatment, and there was also a relationship with sucrose dose. In contrast, there was no significant change in body weight, also probably because of the short administration period, and the results were similar to those of previous experiments. Sweeteners can contribute to diarrhea to some extent by affecting the structure of the intestinal microbiota (Boontiam et al., 2022; Mendoza-Martínez et al., 2022), and excessive sucrose intake may increase the risk of metabolic diseases (Jamar et al., 2021; Sung et al., 2022). In this experiment, although all the treatment groups were able to stop the diarrhea of AAD mice and restore the fecal water content to a normal level, it was obvious that the group with added sucrose was less effective in the recovery of AAD mice than the group with only QWBZP, which was consistent with the results of previous experiments. The organ index is often used to reflect the functional status of the internal organs of animals, and its changes can reflect the effects of food intake and drug interference on the organism, providing some reference data for experimental zoological studies. Liver, thymus, and spleen indices can visually reflect changes in the body and cellular immunity levels. They can roughly estimate the strength of immune function, but it is a crude and lagging indicator. In this experiment, each treatment group was able to improve the liver index and restore it to a normal level but could not improve the spleen and thymus indices, and none of them were significantly different. However, this could not indicate that it has no significant effect on the immune function of mice. After all, this is a rough indicator, which should be discussed and analyzed in conjunction with other results.

Acetic acid, propionic acid, and butyric acid are the most abundant SCFAs in the intestine. As one of the primary metabolites of the intestinal microbiota, SCFAs can lower intestinal pH to a certain extent, thereby inhibiting the viability of pathogenic microbiota from promoting intestinal microecological balance and maintaining the integrity of the intestinal mucosal barrier (Li C. et al., 2022). It can also inhibit the expression of inflammatory factors or alleviate inflammation (Ran et al., 2018). In this experiment, the content of acetic acid, propionic acid, and butyric acid were downregulated by antibiotic modeling, with a significant difference in propionic acid and butyric acid, and the content of total SCFAs was also downregulated. After treatment with QWBZP, the content of acetic acid, propionic acid, and butyric acid were upregulated, with a significant difference in propionic acid, and the content of total SCFAs was restored to a normal level. After treatment with sucrose in QWBZP, the content changes of propionic acid and butyric acid had significant differences. It could be speculated that the addition of sucrose also had a certain correlation with propionic acid and butyric acid.

IL-17 is an inflammatory cytokine produced by activated T cells, which can lead to inflammation by promoting T cell activation and stimulating epithelial cells to create a variety of cytokines, further aggravating diarrhea symptoms. IL-17 plays a crucial role in the fight against intestinal infections, mediates intestinal inflammation and immune response in infectious diarrhea, and is closely related to chronic infectious diarrhea. Widespread and persistent intestinal inflammation is the pathological basis of diarrheal disease. It has been found that colitis caused by Clostridium difficile infection, especially after the use of antibiotics, effectively induces IL-17 production in mice intestinal γδT cells. In contrast, SCFAs, especially propionate, inhibit IL-17 production in IL-17-producing γδT cells (Chen et al., 2020; Dupraz et al., 2021). It was shown that short-term high sugar intake significantly alters the composition of the intestinal microbiota, while long-term sustained high sugar intake induces an intestinal inflammatory response and that high sugar intake does not exacerbate the inflammatory response in antibiotic-treated mice (Jamar et al., 2021). In this experiment, it was concluded that the IL-17 content was significantly downregulated and restored to a normal level after treatment with QWBZP. The IL-17 content was higher in the groups with different sucrose dose, which was consistent with previous studies results. However, sucrose was given along with QWBZP treatment in this experiment, which is somewhat different from the direct administration of a high-sugar diet in previous studies, and the period of this experiment was short, so further research is needed to determine whether the addition of sucrose during treatment can cause intestinal inflammation.

The intestinal mucus layer is part of the intestinal mucosal barrier, which is also the first line of defense of the intestinal mucosal barrier, and plays an essential role in maintaining intestinal homeostasis (Frede and Milling, 2021). Glycoproteins, the primary gel substance in the mucus layer, enhance the mucus barrier effect and alleviate intestinal inflammation (Mei et al., 2019). Mucin is the primary glycoprotein that constitutes the intestinal mucosal barrier, with MUC2 being the most secreted mucin in the gastrointestinal tract, maintaining the integrity of the mucus barrier, which is closely related to intestinal microbiota homeostasis (Henrick et al., 2021). MUC2 is anchored in enteroendocrine cells after secretion, and damage to enteroendocrine cells exacerbates diarrhea (Wen et al., 2019). In this experiment, the treatment with QWBZP significantly upregulated MUC2 content and restored it to a normal level. The MUC2 content was lower in the groups with different sucrose dose, which is consistent with the above conclusion that high glucose intake causes inflammatory reactions.

A variety of normal microbiota are present in the animal intestine, and these commensal microbiotas form a biological barrier by adhering or binding to the intestinal mucosa. Normal microbiotas settle and reproduce in the animal’s internal environment, maintaining a symbiotic relationship with the host and providing the host with nutrients to maintain the intestinal microecological balance. In this experiment, from the Alpha diversity, Beta diversity, and OTU number, it could be found that antibiotic modeling changed the intestinal microbiota structure, while that gradually recovered to a normal level after treatment with QWBZP. Sucrose intake changed the intestinal microbial species and microbiota diversity, which affected the recovery of intestinal microbiota structure. The bacterial microbiome of the human gut at the phylum level is generally dominated by the Firmicute and Bacteridota, which typically account for 80–90%. Proteobacteria is a Gram-negative bacterial phylum. It can contribute to the production of large amounts of pro-inflammatory cytokines and usually maintained at a low level of relative abundance in the gut, which can be used as a marker of whether the intestinal microbiota is dysbiosis (Shin et al., 2015; Rizzatti et al., 2017). In this experiment, the relative abundance of Firmicutes and Bacteroidota showed a decreasing trend in the intestinal microbiota after antibiotics modeling, while the relative abundance of Proteobacteria showed an increasing trend. However, QWBZP intervention caused an increase in the relative abundance of Firmicutes and Bacteroidota in AAD mice and inhibited the growth of Proteobacteria, restoring their relative abundance to normal levels; the intake of different sucrose dose affected the relative abundance of Firmicutes, Bacteroidota, and Proteobacteria. However, this experiment had no significant differences in the above phyla changes. We discussed the top 15 genera and found that the antibiotic modeling significantly downregulated the relative abundance of unclassified_*Lachnospiraceae*, *Bacteroides*, *Faecalibacterium*, *Prevotella*_9, unclassified_*Muribaculaceae*, *Dialister*, *Megasphaera*, *Subdoligranulum*, unclassified_*Bacilli*, *Ruminococcus*, *Alloprevotella*, *Agathobacter*, and significantly upregulated the relative abundance of *Escherichia_Shigella*, suggesting that the above genera play a negative or positive role in the development of diarrhea. After treatment with QWBZP, the relative abundance of *Escherichia_Shigella* was significantly downregulated, while the relative abundance of the remaining 12 genera was significantly upregulated. Compared to the treatment with QWBZP, the relative abundance of most genera was significantly downregulated by the sucrose addition, suggesting that sucrose intake can lead to structural changes in the main dominant genera and a decrease in beneficial bacteria.

Line discriminant analysis effect size (LEfSe) analysis can reveal microbial markers in each group. In this experiment, by analyzing the correlation between the characteristic genera, the inflammatory factor IL-17, mucosal protective factor MUC2, and SCFAs, it was found that, among the characteristic genera enriched in the N group, *Megasphaera*, unclassified_*Lachnospiraceae* showed a significant positive correlation with IL-17 and a significant negative correlation with SCFAs. Among the characteristic genera enriched in the M group, unclassified_*Xanthobacteraceae* showed a significant positive correlation with IL-17 and a significant negative correlation with SCFAs; *Prevotellaceae*_NK3B31_group showed a significant positive correlation with IL-17 and a significant negative correlation with MUC2 and SCFAs. Among the characteristic genera enriched in the Q group, [*Eubacterium*]_*ventriosum*_group showed a significant negative correlation with IL-17 and a significant positive correlation with MUC2 and SCFAs; *Solibacillus* showed a significant positive correlation with MUC2 and SCFAs; [*Ruminococcus*]_*gauvreauii*_group, *Enterococcus* showed a significant negative correlation with IL-17 and a significant positive correlation with SCFAs; Bacterial biomarkers may indicate the role of QWBZP in AAD. The characteristic genera enriched with different sucrose dose groups were significantly positively or negatively correlated with different SCFAs, while there was no significant correlation with IL-17 and MUC2. These conclusion were consistent with the previous results concerning the content changes of IL-17, MUC2, and SCFAs in each group.

Microbial biomass and microbial activity are typical indicators for studying the characteristics of microbial communities in natural samples (Tao et al., 2021; Wu et al., 2021), and the effect or regulation of intestinal microecology by pharmaceutical interventions can be assessed to some extent by detecting changes in both. The method of reflecting the level of microbial activity by the activity of FDA hydrolase was initially applied to soil microbiota (Jiang et al., 2011). We have matured to use the FDA method to evaluate the overall activity of animal or human intestinal microbiota. In this experiment, the fecal microbial activity was significantly reduced after antibiotics modeling. It can be speculated that antibiotics inhibit the growth of certain microbiota. In contrast, the fecal microbial activity increased significantly to normal levels after treatment, which is consistent with the changes in microbial biomass in the previous section.

The intervention of AAD with QWBZP effectively reduced harmful intestinal bacteria and increased beneficial intestinal bacteria and SCFAs, which may be why it affected intestinal microbiota function. Still, the exact mechanism needs to be further investigated. PICRUSt2 functional prediction revealed that different sucrose dose caused significant changes in the functions of intestinal microbiota mainly in glycolysis/gluconeogenesis, pentose phosphate pathway, purine metabolism, pyrimidine metabolism, starch and sucrose metabolism after treatment with QWBZP, and further studies on the functional aspects of these intestinal microbiotas are needed in the future.

## 7. Conclusion

Based on the previous study, the dose of the sweetener sucrose was discussed in this experiment. It was found that the addition of sucrose had an effect on the efficacy of the AAD intervented by QWBZP, which was less effective than QWBZP, showing a certain dose-response relationship. In this experiment, it was concluded that the addition of sucrose might also further lead to intestinal inflammation and the disruption of the intestinal mucosal barrier, and the production of metabolites SCFAs. However, these findings still need to be verified in a more extensive study. The effect of adding the sweetener sucrose on the efficacy of Chinese herbal medicine in treating diseases also still needs more research.

## Data availability statement

The datasets presented in this study can be found in online repositories. The names of the repository/repositories and accession number(s) can be found in the article/supplementary material.

## Ethics statement

This animal study was reviewed and approved by Institutional Animal Care and Use Committee of the Hunan University of Chinese Medicine.

## Author contributions

ZT designed the study. CL wrote the manuscript, analyzed the data, and performed the experiments. NX and ND analyzed the data. NX, DL, ND, and ZT supervised the work and reviewed the manuscript. MP provided funding sources. All authors contributed to the article, decided to submit the manuscript for publication, and approved the submitted version.
